# Introduction to the United Kingdom National Multidisciplinary Guidelines for Head and Neck Cancer

**DOI:** 10.1017/S0022215116000359

**Published:** 2016-05

**Authors:** V Paleri, N Roland

**Affiliations:** 1 Department of Otolaryngology-Head and Neck Surgery, The Newcastle Upon Tyne Hospitals NHS Foundation Trust, Northern Institute of Cancer Research, Newcastle Upon Tyne, UK; 2 Department of Otolaryngology-Head and Neck Surgery, Aintree University Hospitals NHS Foundation Trust, Liverpool, UK

## Abstract

This is the 5th edition of the UK Multi-Disciplinary Guidelines for Head and Neck Cancer, endorsed by seven national specialty associations involved in head and neck cancer care. Our aim is to provide a document can be used as a ready reference for multidisciplinary teams and a concise easy read for trainees. All evidence based recommendations in this edition are indicated by ‘(R)’ and where the multidisciplinary team of authors consider a recommendation to be based on clinical experience, it is denoted by ‘(G)’ as a good practice point.

It is an enormous privilege and a great pleasure to introduce the 5th edition of the UK Multi-Disciplinary Guidelines for Head and Neck Cancer. Akin to the 4th edition,^1^ each aspect of the guideline has been developed by an expert team, often multidisciplinary. An affirmation of the true multidisciplinary nature of these guidelines is the endorsement by seven medical specialty organisations involved in head and neck cancer care in the UK: British Association of Endocrine and Thyroid Surgeons, British Association of Head and Neck Oncologists, British Association of Oral and Maxillofacial Surgeons, British Association of Otorhinolaryngology-Head and Neck Surgery, British Association of Plastic, Reconstructive and Aesthetic Surgeons, The Royal College of Pathologists and The Royal College of Radiologists (Faculty of Clinical Oncology). The guidelines will be of interest across the spectrum of healthcare professionals who look after patients with Head and Neck Cancer.

Our aim was to produce multidisciplinary consensus recommendations on the management of Head and Neck cancer based on the expertise and experience invested within the UK-based international experts and their appraisal of the current evidence. The remit of these guidelines is to provide evidence-based recommendations that will help identify an optimal management strategy. It should be appreciated that the ultimate decision for the management should rest with the multidisciplinary team, which takes into account all clinical data pertaining to the patient and his or her own social circumstances and individual preferences.

In contrast to the 4th edition, we have migrated away from the Scottish Intercollegiate Guidelines Network (SIGN) grading of recommendations. In 2013, SIGN abandoned its ABCD grading method^2^ as it became evident that not all research would fit within the constraints of this system. Scottish Intercollegiate Guidelines Network has since adopted the system developed by the Grading of Recommendations Assessment, Development and Evaluation (GRADE) working group.^3^ Having studied the GRADE methodology in detail, we concluded that a guideline such as this, generated by a multidisciplinary group of practising clinicians, simply did not possess the resources and the time to use the GRADE methodology. Similar to some of the more recent SIGN guidelines, all evidence-based recommendations in this edition come without a grade attached, indicated by ‘(R)’ and where the multidisciplinary team of authors consider a recommendation to be based on clinical experience, it is denoted as a good practice point ‘(G)’.

The 5th edition will again provide a robust clinical document, which can be used as a ready reference. and a concise easy read for trainees and all involved in Head and Neck cancer care. In conjunction with the upper aerodigestive tract cancer guidelines published recently by the National Institute for Health and Care Excellence,^4^ the recommendations across these two publications should improve the care provided to this complex disease. The tremendous amount of work put in by the authors is being recognised by individually indexed publications; however, we would recommend that readers use this supplement in the Journal of Laryngology and Otology as a single document owing to the cross-referencing within it. We are confident that the publication of the 5th edition as a journal supplement will enhance readership and facilitate greater dissemination across the Head and Neck community.
